# Interaction Networks Help to Infer the Vulnerability of the Saproxylic Beetle Communities That Inhabit Tree Hollows in Mediterranean Forests

**DOI:** 10.3390/insects14050446

**Published:** 2023-05-09

**Authors:** Javier Quinto, Cecilia Díaz-Castelazo, Alfredo Ramírez-Hernández, Ascensión Padilla, Esther Sánchez-Almodóvar, Eduardo Galante, Estefanía Micó

**Affiliations:** 1 Instituto de Investigación CIBIO (Centro Iberoamericano de la Biodiversidad), Universidad de Alicante, 03690 Alicante, Spain; ma.padilla@ua.es (A.P.); galante@ua.es (E.G.); e.mico@ua.es (E.M.); 2 Instituto de Ecología A.C. (INECOL), Veracruz 91073, Mexico; diazcastelazogm@gmail.com; 3 CONACYT/IPICYT—División de Ciencias Ambientales, San Luis Potosí 78216, Mexico; alfredo.ramirez@ipicyt.edu.mx; 4 Instituto Interuniversitario de Geografía, Universidad de Alicante, 03690 Alicante, Spain; esther.sanchez@ua.es

**Keywords:** beta diversity of interactions, conservation, insect decline, interaction decline, network analysis, network stability, temporal shifts

## Abstract

**Simple Summary:**

Insect populations are facing unprecedented changes in many ecosystems worldwide. However, do these changes make insect communities more vulnerable? The study of interaction networks can help to answer this question. We assessed the adequacy of network tools to address the long-term variation (after 11 years) of diversity patterns of the saproxylic (wood-dependent) beetle communities that inhabit tree hollows in three representative Mediterranean woodland types. To explore saproxylic communities’ vulnerability to microhabitat loss, we simulated hollow extinctions and recreated feasible future threat scenarios based on decreasing microhabitat suitability. Contrasting responses in diversity patterns among woodland types were found, whereas interaction patterns generally showed substantial temporal variations in the way that saproxylic beetles interact with tree hollows (less interconnected and specialized networks). Network procedures evidenced increased saproxylic communities’ vulnerability, and this situation could worsen in potential future scenarios with decreased microhabitat suitability. The valuable information that ecological networks provide should be considered for improving management and conservation programs.

**Abstract:**

Insect communities are facing contrasting responses due to global change. However, knowledge on impacts of communities’ reorganizations is scarce. Network approaches could help to envision community changes in different environmental scenarios. Saproxylic beetles were selected to examine long-term variations in insect interaction/diversity patterns and their vulnerability to global change. We evaluated interannual differences in network patterns in the tree hollow–saproxylic beetle interaction using absolute samplings over an 11-year interval in three Mediterranean woodland types. We explored saproxylic communities’ vulnerability to microhabitat loss via simulated extinctions and by recreating threat scenarios based on decreasing microhabitat suitability. Although temporal diversity patterns varied between woodland types, network descriptors showed an interaction decline. The temporal beta-diversity of interactions depended more on interaction than on species turnover. Interaction and diversity temporal shifts promoted less specialized and more vulnerable networks, which is particularly worrisome in the riparian woodland. Network procedures evidenced that saproxylic communities are more vulnerable today than 11 years ago irrespective of whether species richness increased or decreased, and the situation could worsen in the future depending on tree hollow suitability. Network approaches were useful for predicting saproxylic communities’ vulnerability across temporal scenarios and, thus, for providing valuable information for management and conservation programs.

## 1. Introduction

Insect communities are currently facing serious and contrasting changes across taxa and spatial scales as a consequence of global change. There is growing evidence that many insect populations are declining, although positive or neutral responses (population increases or stasis) have also been reported [1,2,3,4,5,6]. Insect decline involves not only rare or charismatic species (specialists), but also historically very common or generalist species [7,8,9,10,11] that play an irreplaceable role in the provision of important ecosystem services [12,13], such as nutrient recycling, pollination, control of insect populations, and a key contribution to the maintenance of food chains. Anticipating the consequences of temporal changes in insect communities is, therefore, a central challenge because environmental health and economy depend to a large extent on the ecosystem services that they provide [13,14,15,16,17]. Moreover, the success of integrative conservation strategies implies considering insect ecological requirements (habitats/microhabitats)—commonly underrepresented in large-scale conservation assessments and protected area designation [18,19]—as well as their potential alteration in the current global change context.

There is a general consensus that the stressors of global change (i.e., habitat loss or land degradation, agricultural intensification, invasive species, environmental pollutants, and the manifold impacts of climate change [8,11,14,20,21,22]) create “winners” and “losers”. However, information on the consequences of the undisputed reorganization of insect communities in ecological terms is still scarce [4,11,12,23]. Most studies that address long-term changes in insect communities consider variations in species richness and abundance over time [6,9,14,15,16,20,24,25], which limits the ability to explain response mechanisms to environmental changes and to estimate their vulnerability in different environmental scenarios [26]. There is an urgent need for studies that dig more deeply into temporal changes from different approaches to take a step forward in the understanding of current population changes and in the prediction of future trends [27]. Hence, network analysis approaches can complement taxonomic diversity tools to properly answer these questions and to envision community changes in plausible environmental scenarios.

Ecological networks not only describe the interactions between species and the underlying structure of communities but also exhibit emergent properties on ecosystem function and stability [28]. Therefore, the temporal approach to the study of interaction networks can help to identify the ecological factors that lie behind population changes and to figure out the ecological consequences of changes in species diversity and interaction patterns [29,30,31,32]. Additionally, integrating beta diversity to understand the turnover in the diversity of interactions between networks can help to determine whether temporal changes are due to changes in species composition or to changes in interaction turnover [33,34,35].

Furthermore, examining the temporal changes in the network’s robustness to species extinctions is promising because it can help to recognize species that are disproportionately important for not only the network’s integrity but also for insect communities’ resilience [35,36]. As a step forward, the simulation sequences of microhabitat loss enables us to reflect on the risk of ecological communities’ extinction when faced with potential threat scenarios [37,38]. 

One model group to check the extent of ecological network tools to examine long-term variations of insect populations and their vulnerability to global change is saproxylic (dead-wood-dependent) beetles. These beetle communities are extremely diverse and exploit different forest habitats and microhabitats [39,40]. Of forest microhabitats, tree hollows are particularly relevant for being keystone structures that maintain forest biodiversity in Europe [41,42,43]. In addition, tree hollows host specialist species with low dispersal ability and narrow geographic ranges (low turnover rates), including endangered species that can be particularly vulnerable to global threats [44]. Tree hollows can be surveyed with emergence traps, a standardized absolute method that allows quantitative abundance monitoring over time (i.e., complete years) [45], and it avoids one of the commonest shortcomings that occurs when evaluating long-term insect population variations [46]. Finally, this community is very sensitive to some of the main global stressors, namely land use changes, increasing temperatures, or climate-induced changes in natural disturbance regimes [8,47,48,49]. 

With this background, the general aim of this study was to assess long-term variations in saproxylic beetle communities linked with tree hollows to predict their vulnerability in the Mediterranean region, where the impact of the global warming process is particularly pronounced [50,51]. We evaluated interannual differences in the network topology and interaction patterns in the tree hollow–saproxylic beetle interaction by conducting two absolute 1-year samplings separated by 11 years in three Mediterranean woodland types nestled in the protected Cabañeros National Park (central Spain). Studies in protected or remote areas are a priori less susceptible to anthropogenic impacts, such as land use change [48,52,53] and, thus, allow a more refined analysis about the impacts of environmental pollutants or climate trends.

We specifically address the following questions: (1) Do network structure and species diversity patterns of the tree hollow–saproxylic interaction differ over time? (2) To what extent are changes in the tree hollow-saproxylic interaction patterns explained by changes in species composition vs. interaction turnover? (3) How vulnerable are saproxylic communities in protected areas in the current global change context? (4) How could they evolve in the near future? We hypothesized that tree hollow–saproxylic insect networks in Mediterranean woodland types would be nested as reported in [54], and this specialized interaction pattern would tend to remain constant in spite of the high temporal turnover in species composition and interactions [29,31,32,55]. Nonetheless, major changes in the interaction properties of tree hollow–saproxylic networks are expected due to foreseeable temporal shifts in species diversity and species interactions reported in natural ecosystems in general—e.g., [56]—and in saproxylic communities in forest ecosystems in particular [2,49]. Losses in species richness and abundance, and the inherent in-depth restructuring of interactions that they entail, are expected to provoke bottom-up cascading effects on the tree hollow–saproxylic food web that will lead to increased vulnerability, e.g., [13,15,57]. We hypothesize that network tools will be extremely useful for looking ahead with the recreation of feasible threat scenarios based on the elimination of the most suitable tree hollows [58]. Understanding long-term variation in the structure and stability of saproxylic networks is a new and promising approach that will help to develop more accurate forest management and conservation programs to mitigate insect decline.

## 2. Materials and Methods

### 2.1. Study Area and Data Collection

**Figure 1:**
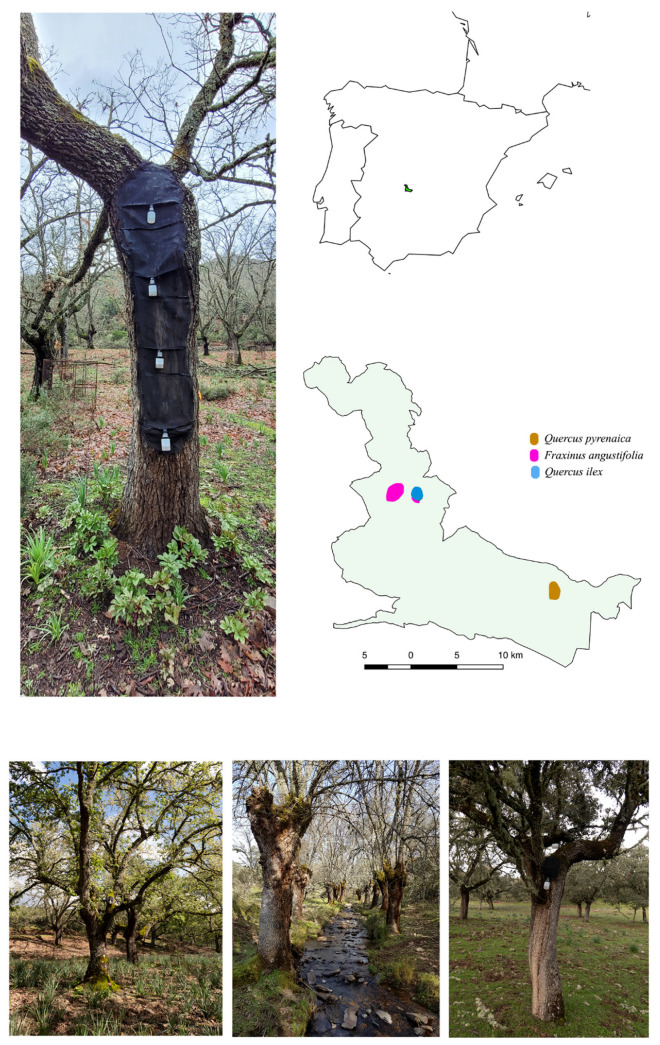
Details of the study area. Emergence trap. Location of the study area in central Spain. Location of the studied woodland types in the Cabañeros National Park. *Quercus pyrenaica* woodland. (*Fraxinus angustifolia* woodland. *Quercus ilex* woodland.

The study was carried out in the Cabañeros National Park (central Spain) (Figure 1), a large natural area of 40856 ha devoted to protecting and preserving Mediterranean ecosystems. Since it was declared a National Park in 1995, there has been no serious human interference [59]. The National Park comprises a limited number of mature woodlands embedded in a predominantly grassland and scrubland matrix. Three of the most representative Mediterranean woodland types in the park were considered for this study: (i) a deciduous oak woodland of *Quercus pyrenaica* Willd. (39°21′20.37″ N, 4°23′42.15″ W), in which elevation ranges from 732 to 774 m; (ii) a deciduous riparian ash woodland of *Fraxinus angustifolia* Vahl. (39°26′50.34″ N, 4°33′49.32″ W) with elevation ranging from 497 to 662 m; and (iii) a sclerophyllous oak woodland of *Quercus ilex* Lam. (39°26′45.48″ N, 4°31′51.90″ W) whose elevation range goes from 658 to 673 m (Figure 1). 

The saproxylic beetle fauna that inhabits tree hollows was surveyed from March 2009 to February 2010 (henceforth t_1_) and March 2021 to February 2022 (henceforth t_2_). Sixteen tree hollows were selected in each woodland type per period. For the selection of tree hollows in t_2_, neighboring woodland patches were considered, and the previously sampled tree hollows were excluded to avoid any effects deriving from fauna extraction during the first sampling (t_1_). A similar pool of different tree hollows was selected during both t_1_ and t_2_, following criteria such as height to the ground, hollow volume, similar proportion of tree hollows at the base, branches and trunk, as well as other abiotic and biotic factors [39,41,60]. Emergence traps were used as the sampling method (Figure 1) and are a very accurate method for monitoring the saproxylic communities that inhabit tree hollows [45]. After installing traps, only the species that came from inside tree hollows could be captured. Samples were collected monthly over 1 year. 

For this study, we considered the whole saproxylic beetle fauna (excluding Cryptophagidae, Latridiidae and Staphylinidae), namely those species with a well-known saproxylic biology [61,62,63] (Table S1). Specimens were deposited at the CEUA-CIBIO collection, University of Alicante, Spain.

### 2.2. Climate Trends

**Figure 2:**
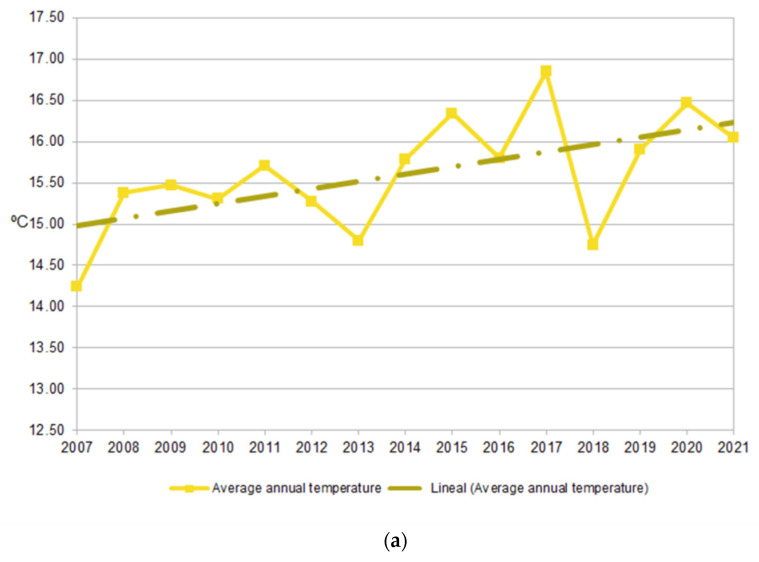

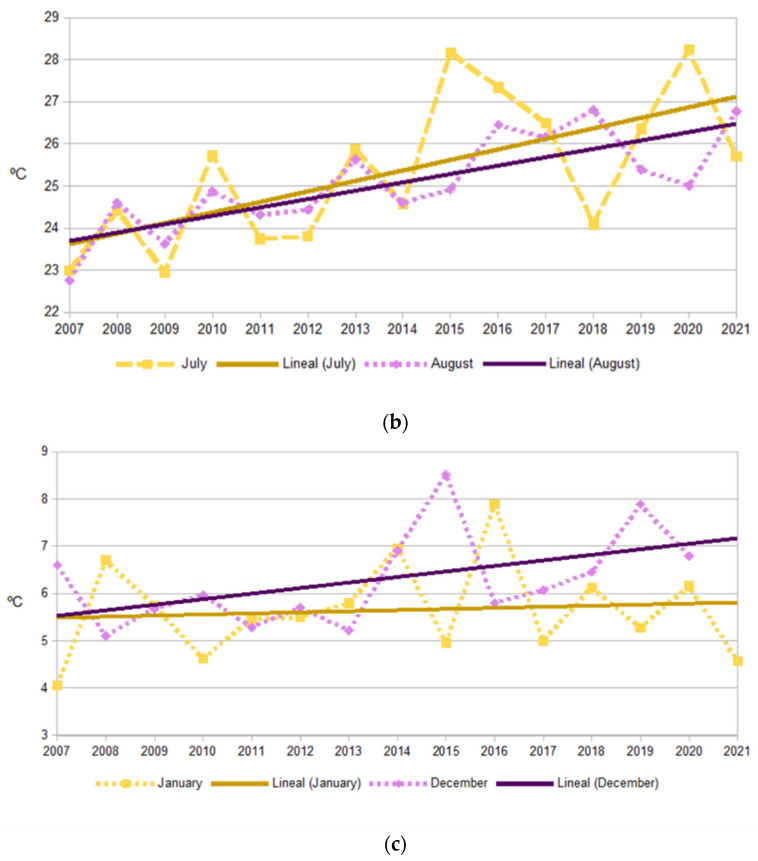
Historical interannual changes in temperature in Cabañeros [AEMET, 2022]: (**a**) average annual temperature and its linear trend; (**b**) average annual temperatures of the warmest months (July and August) and their linear trends; (**c**) average annual temperatures of the coldest months (December and January) and their linear trends. Note the variation in scale on the vertical axes.

We obtained the historical yearly variation from 2007 to 2021 in both the average annual temperature and the average annual temperature in the warmest and coldest months from the closest and most accurate AEMET weather station (Spanish State Meteorological Agency) (Cabañeros National Park PCA01) [64], which is part of the Global Change Monitoring Network (GCMN) of the Spanish National Parks Network. Figure 2a shows how the average annual temperature has increased by more than 1 °C between t_1_ and t_2_. This trend was even more evident when we looked at the average annual temperatures of the warmest months, which reached 3 °C higher in July (Figure 2b). In contrast, the general trend for the coldest months—January and December—remained similar or was slightly lower (Figure 2c). The team is aware that further specific work on this issue is needed.

### 2.3. Diversity Analysis

We assessed temporal variation in diversity patterns between t_1_ (2009–2010) and t_2_ (2021–2022) by considering two analysis levels—the National Park scale and woodland type—because contrasting spatial scales can provide complementary information [45,54]. We first assessed the inventory completeness for each year and for woodland type per year using a sample coverage estimator (*Ĉm*), which is one of the least biased estimators of sample completeness [65]. This estimator ranges from 0 (minimal completeness) to 100% (maximum completeness). To evaluate temporal changes in diversity patterns on the National Park scale and per woodland type, we employed Hill numbers *^q^D* of orders ^0^*D* and ^1^*D* [66]. ^0^*D* represents species richness and is not sensitive to abundances, while ^1^*D* corresponds to ecological diversity and uses the inverse of the exponential of Shannon’s entropy to estimate effective species by weighting the abundance of each species in the sample without favoring either common or rare species [66,67]. Then, the observed *^q^D* values were compared utilizing 95% confidence intervals (95%CI), where non-overlapping 95% CI values indicate significant differences [68]. Such analyses were performed in the iNEXT package v2 [69] in the R software v.4.0.3 [70]. 

Additionally, differences in species composition were evaluated using the Bray–Curtis Index calculated for the samples with a permutational multivariate analysis of variance (PERMANOVA) after 999 permutations of residuals in a simplified model. Then pairwise tests were applied to assess temporal changes for the National Park and per woodland type. Multidimensional scaling (MDS) was used to graph samples’ relative positions according to their similarity in species composition. Bootstrap was the procedure followed for this analysis and to draw 95% confidence ellipses. Both PERMANOVA and MDS were performed using PRIMER v7 [71]. 

### 2.4. Network Patterns and Interacting Attributes

The existence of specialized patterns in tree hollow–saproxylic beetle networks in Mediterranean woodlands has been previously reported, and both the National Park and woodland scale matter [45,54]. We analyzed the interannual variation in specialized network patterns and the potential turnover or reorganization of interactions in the tree hollow–saproxylic beetle interaction on both spatial scales. To calculate nestedness (*NODF*), we performed CE null models in ANINHADO [72]. This null model contemplates the probability of an interaction being proportional to the generalization level (abundances) of tree hollows and saproxylic beetles [73]. This procedure is considered the best available estimation of nestedness because it is based on the nestedness of all pairs of rows and columns in the matrix [74]. Additionally, we used MODULAR [75] to assess modularity with a simulated annealing algorithm to maximize the modularity index (*M*). This procedure provides the most precise modularity estimation because it allows the optimal partition with the largest modularity of any ecological network in modules to be defined [76]. 

The interannual differences in the interacting attributes at the National Park and per woodland type were evaluated, and the basic network graphs were plotted using the bipartite package [77] in the R software v.4.0.3 [70]. The following interacting and ecological indices (at the network level) were evaluated: degree (sum of links per species), species strength (sum of dependencies per species), connectance (proportion of made links of the total possible in each network), links per species (a qualitative measure defined as the sum of links divided by the number of species), linkage density (a quantitative measure defined as the mean number of interactions per species), interaction evenness (homogeneity of interaction frequencies across all the links in the network), specialization index *H2*′ (a network-level measure of specialization ranging from 0 (no discrimination) to 1 (complete discrimination)), and the V-ratio (variance ratio of species numbers to individual numbers within species for the higher trophic level. Values over 1 indicate positive aggregation or co-occurrence; values between 0 and 1 denote disaggregation, competence, or differential microhabitat associations).

### 2.5. Interannual Beta Diversity of Interactions

Interaction turnover between years was explored by analyzing the overall dissimilarity interactions between networks (*β_WN_*), which considers saproxylic beetle abundance and affects the probability of interactions [78]. We calculated beta diversity due to species composition (*β_S_*), beta diversity of interactions in co-occurring species (*β_OS_*) and dissimilarity of interactions due to species turnover (*β_ST_*). *β_WN_* was calculated with this equation: *β_WN_* = *β_ST_* + *β_OS_* [79]. It should be pointed out that *β_ST_* is strongly constrained by *β_S_* values, which means that it could only be really meaningful when *β_S_* takes intermediate values (close to 0.5). The betalink and igraph packages [79] in the R software v.4.0.3 [70] were employed to calculate all the beta measurements.

### 2.6. Microhabitat Loss Simulations

Using the bipartite package, we also explored vulnerability to species extinctions (functionrobustness) by addressing the three available scenarios of simulated tree hollow loss as the level of saproxylic beetle communities’ tolerance to loss [37] or the severe alteration of their microhabitats. This function calculates the area below the extinction curve generated by the removal of tree hollows, where R = 1 corresponds to a curve that decreases very slightly up to the point at which almost all tree hollows are eliminated, whereas with R = 0, the curve sharply decreases as soon as any tree hollow is removed [80]. First, tree hollows were randomly removed; second, a sequence of elimination from the most to the least connected tree hollows was followed; lastly, the tree hollows with less beetle abundance were first eliminated. These three approaches were considered to obtain complementary information about the hypothetical processes that would lead to microhabitat loss. 

In addition, we delved into the ecological role of core tree hollows for tree hollow–saproxylic networks’ stability. Core–periphery structures are rather complementary measurements of network topology and stability [54] and often identify a core of densely and heterogeneously interconnected generalist species surrounded by sparsely linked specialists on the periphery [81]. At first glance, a marked decline in beetle abundance in the study area in only 11 years was easily noted. Thus, the core composition could have been temporarily and critically affected, and—most importantly—this trend could worsen with time. For this purpose, qualitative and quantitative core/periphery approaches were examined to compare both the number and identity of the tree hollows along the core–periphery gradient between t_1_ and t_2_. We first identified qualitative tree hollow cores with the equation proposed by Dáttilo et al. [82], *Gc* = (k_i_ − *k_mean_*)/ *σ_k_*, where k_i_ corresponds to the number of links for a given tree hollow/beetle species, *k_mean_* is the mean number of links for all the tree hollow/beetle species in the network, and *σ_k_* is the standard deviation of the number of links for tree hollow/beetle species. Only those nodes with *Gc* > 1 are constituents of the generalist core. Second, to identify quantitative tree hollow cores (based on interaction frequency data), we used the species-level parameter of “species strength” (bipartite package), an index that measures the relative importance of a node at a given guild/partition/trophic level for the counterpart (the nodes at the other guild/partition/trophic level) [83,84]. We established species strength values above 4 as an arbitrary benchmark to quantitatively consider a node to be a core component because the values of this attribute in our study sharply dropped below this mark. In light of the foregoing, we proposed two potential threat scenarios for saproxylics based on the alteration of core nodes. The degree values in the original interaction matrix for tree hollows were modified so that the core tree hollows were transformed into less suitable ones by giving them the worst values in species composition (those tree hollows with lower degree values) based on (a) qualitative core transformation and (b) quantitative core transformation. Finally, we followed simulated extinction tree hollow microhabitat procedures, as previously mentioned.

## 3. Results

### 3.1. Long-Term Variation in Diversity Patterns

**Figure 3:**
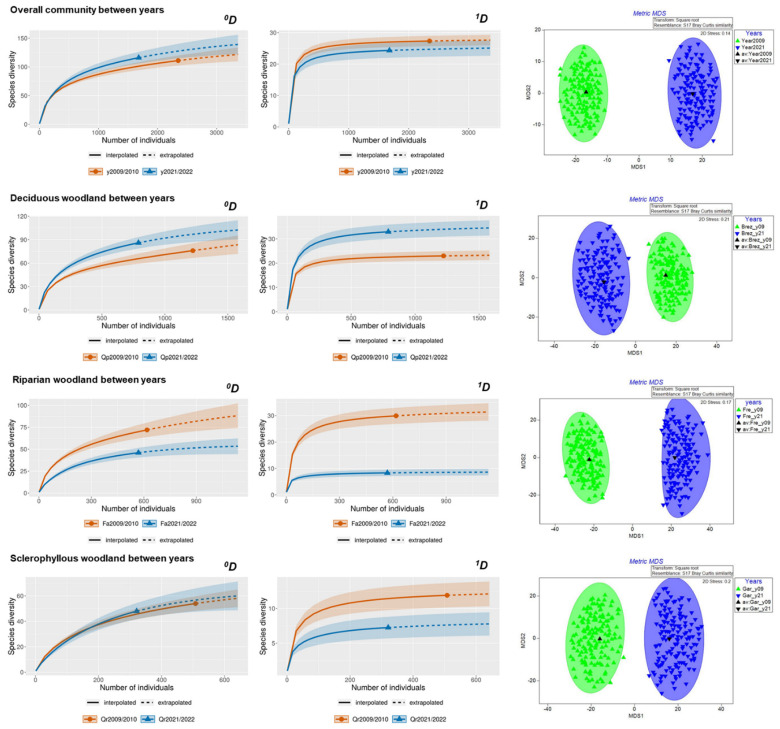
Differences in species richness (^0^*D*) (left), ecological diversity (^1^*D*) (middle), and species composition (right) of saproxylic beetles between years on the park scale (overall community) and the woodland scale. Note the variation in scale on the vertical axes.

In all, 153 saproxylic species and 4028 individuals belonging to 34 Coleoptera families were recorded (Table S1). Sample coverage was >93% in all cases (for each forest type and sampling year), which indicates suitable sampling effort. We did not detect differences in species richness or ecological diversity between t_1_ and t_2_ on the park scale, but we obtained contrasting responses on the woodland scale (Figure 3). No differences in species richness were found in the deciduous oak woodland, while ecological diversity ^1^*D* was higher in t_2_. In the riparian woodland, both species richness and ecological diversity decreased significantly in t_2_. In the sclerophyllous woodland, no differences in species richness were observed, but differences appeared for ecological diversity, which was lower in t_2_. Major interannual changes in species composition were detected in all cases: park scale (F_pseudo_ = 4.59, df = 1, *p* = 0.001), deciduous oak woodland (F_pseudo_ = 2.05, df = 1, *p* = 0.011), riparian woodland (F_pseudo_ = 3.68, df = 1, *p* = 0.001), and sclerophyllous woodland (F_pseudo_ = 2.06, df = 1, *p* = 0.006) (Figure 3).

### 3.2. Interannual Changes in Network Patterns and Interacting Attributes

**Figure 4:**
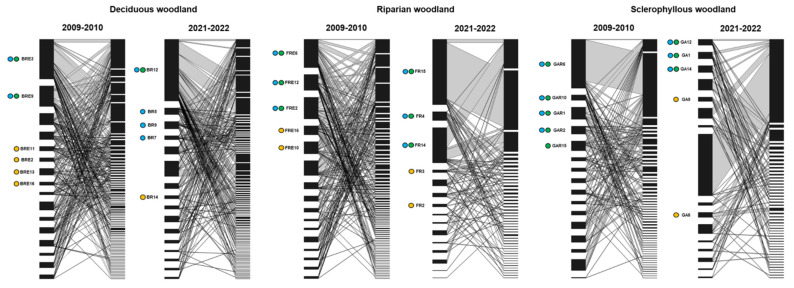
Bipartite graphs representing the diversity of interactions between tree hollows (left side) and saproxylic beetles (right side) between years per woodland type. Core tree hollows are marked with circles: qualitative cores in green, the quantitative cores with stronger interaction strength than 5 in blue, and the quantitative cores whose interaction strength lies between 4 and 5 in yellow.

A significant nested pattern was found on the park scale across the years. By disentangling by woodland type, contrasting responses were evidenced (Figure 4, Table 1). The deciduous *Quercus* woodland conserved the nested pattern across the years. The riparian woodland showed nestedness in t_1_ but not in t_2_, and the sclerophyllous woodland did not show a nested pattern in any study year. In all the combinations, the nestedness values from t_1_ to t_2_ clearly dropped (Table 1). Conversely, we did not find modular patterns in any scenario (*p* > 0.05).

Interacting attributes—such as connectance, links per species, and linkage density—considerably decreased on the park scale and per woodland type over time, especially in the riparian woodland, e.g., linkage density decreased to one third in t_2_ (Table 1). Interaction evenness decreased in all cases but particularly in the riparian woodland. Moreover, at all levels, a conspicuous increase took place in network-level specialization (*H2′*). Similarly, the variance ratio showed higher specialization, which indicates an increase in species disaggregation in saproxylic communities and was particularly marked in the riparian woodland.

### 3.3. Network Beta Diversity

The beta diversity of saproxylic beetles between the years (*β_S_*) showed intermediate values (around 0.70) on the park scale and per woodland type (Table 2). The mean temporal beta diversity of the interactions between co-occurring species (*β_OS_*) was 0.20 on the park scale. On the woodland scale, values ranged from 0.3 to 0.45. In contrast, dissimilarity of interactions due to species turnover (*β_ST_*) was always lower (from 0.04 to 0.12). In this vein, interannual changes in interactions (*β_WN_*) were influenced more by interaction turnover or reconnection of interactions (*β_OS_*) than by changes in species composition or species gain/loss (*β_ST_*). This trend was particularly noticeable in both the riparian and sclerophyllous woodlands (Table 2).

### 3.4. Vulnerability to Microhabitat Loss

The study of simulated threat scenarios allowed us to identify interannual differences in saproxylic species’ vulnerability to microhabitat loss suitability. Saproxylic networks showed high sensitivity to loss of the most heterogeneous interconnected tree hollows in t_1_ (RM: 0.61–0.70) and t_2_ (RM: 0.54–0.73) on the park scale and per woodland site (Table 1). The main changes between the years occurred on the park scale and were more sensitive at t_2_. They showed relatively good tolerance to the elimination of the least interconnected tree hollows (RL was almost always around 0.97–0.99) (Table 1). Only in the riparian woodland was a relevant decrease in robustness between years observed (RL_t1_ = 0.98, RL_t2_ = 0.89). The random extinction of tree hollows had an intermediate effect on saproxylic communities’ stability (RR: 0.80–0.93) (Table 1) and revealed a noticeable decline in network robustness, especially on the park scale and in the riparian woodland.

Based on the transformation of the core tree hollows, the study of potential threat scenarios evidenced a marked similarity in the pool of tree hollows that acted as a core in both the qualitative and quantitative approaches, particularly when interaction strength values over 5 were considered (Figure 4). The main exception was the deciduous *Quercus* woodland, where more quantitative cores were found in the quantitative approach (Figure 4). A more heterogeneously distributed group of quantitative core tree hollows appeared when considering species strength values over 4. When simulated extinctions were performed, we found less specialized interaction patterns because the nested pattern only remained on the park scale (it disappeared in the *Q. pyrenaica* woodland in both scenarios). Furthermore, we distinguished a gradual decrease in robustness in the random (RR) and directed (RM and RL) simulated extinctions, which was especially noticeable in both the sclerophyllous and riparian woodlands (Table 1).

## 4. Discussion

This study evidences the usefulness of network tools to forecast saproxylic communities’ ecological vulnerability across temporal scales in the current global change context. The results were partially consistent with what we hypothesized because we found contrasting temporal responses for network structure and interaction properties across different forest types, i.e., nestedness was conserved in the *Q. pyrenaica* woodland over time but was lost in the riparian woodland in t_2_, and no specialized patterns were detected in the sclerophyllous woodland. We also showed marked reorganizations in the network of interactions, which were influenced more by changes in the interaction turnover than by changes in species composition (diversity patterns). Our results revealed that using ecological networks consistently improved the understanding of long-term reorganizations in saproxylic communities and helped to infer their vulnerability in present and future scenarios. All these results contribute to untangling long-term saproxylic dynamics in forest ecosystems.

### 4.1. Interannual Changes in Network and Diversity Patterns

Nestedness was the only network pattern found in the tree hollow–saproxylic beetle interaction. The existence of nestedness in ecological networks implies a tendency for nodes to interact with proper subsets of better-connected nodes [85], the most generalist tree hollows, and beetles. In general, the nested pattern found in tree hollow–saproxylic beetle networks evidences an asymmetry in how insects use tree hollows; consistent pool tree hollows would be more preferred or more suitable and would maintain a high richness of associated interacting species, while others would share few species/interactions [54]. This suggests that the nested structure in saproxylic networks is positively related to habitat and microhabitat complexity [54,86], especially to the availability of suitable tree hollows, defined as the provision of large multihabitat systems with a wide range of microhabitats [87]. In this way, Mediterranean forests housing a larger proportion of suitable tree hollows determine higher saproxylic diversity [39], which leads to more complexity and a higher density of interactions and, ultimately, to a more nested interaction assemblage [54]. The assessment of two spatial sampling scales (park and forest type) revealed that suitable tree hollows act as crucial connectors by promoting the nested pattern across spatial scales and evidenced that more complex woodland types (*Q. pyrenaica* in our case) contribute more to nestedness on the higher spatial scale (park).

Saproxylic beetle communities are temporally dynamic and evenly show wide interannual variation in diversity and composition in Mediterranean forests [42,88]. In contrast, very little is known about how saproxylic networks evolve over time (see [89]). Basic community structure descriptors, such as connectance, links per species, and linkage density, underwent a substantial temporal decrease across the spatial scales in our study system contrarily to [90], which reflects general interannual loss in both diversity and abundance of interactions. It is known that drastic decreases in connectivity (number of interactions) can jeopardize the nested structure [91]. It is also true, however, that temporally dynamic ecological networks are able to preserve nestedness in spite of strong reorganizations in species and interaction diversity, which means that interacting species are gradually replaced by others, maintaining the nested structure [31,32] even across spatial scales [92,93,94]. This is what presumably occurred on the park scale and in the deciduous *Quercus* woodland. However, nestedness was lost in the riparian woodland over time, which suggests that changes in the availability of suitable tree hollows were probably so abrupt that the connectivity and occurrence of nestedness were seriously affected [91,94,95]. Overall, saproxylic assemblages in Mediterranean woodlands are undergoing severe interaction decline and network simplification.

From another point of view, lack or loss of nestedness in combination with high specialization values (as found in the sclerophyllous and riparian woodlands in t_2_) may also point out differential patterns of interactions, e.g., [96], in which very few species were shared throughout tree hollows (disaggregation). Accordingly, loss of nestedness in the riparian woodland would come with increased specialization. What is more, the widest interannual variations in the assessed community structure descriptors occurred in the riparian woodland. For example, the variance ratio revealed a temporal shift from a positive to a negative species association, which clearly highlights interannual changes in the way in which saproxylics interact or select tree hollows for their suitability (they tend to be rarer or specialist). In addition, interaction evenness decreased on all the analysis scales, which indicates homogenization of interactions [97], particularly in the riparian woodland. These results stress that saproxylic networks in Mediterranean riparian forests decline more drastically than other forest types in the long term which, in turn, suggests that the remaining pool of tree hollows would harbor weaker communities because fewer alternatives (suitable tree hollows) are available for such specialist communities. The existence of riparian forests is threatened by global climate change in Mediterranean areas as a consequence of more streams drying out [98]. Moreover, in Cabañeros National Park, saproxylic beetles’ dispersal possibilities in riparian forests are limited by rather low coverage and connectivity [99].

Striking temporal responses in diversity patterns were found, with sclerophyllous and riparian woodlands recording the most marked declines in species richness and ecological diversity. A sharp temporal decline in the richness, abundance, and diversity of forest insects related to global change has been documented [15,48,100,101]. Of global stressors, the most probable causes of insect decline in Cabañeros (inexistence of land use) could be increased pollution and climate change (as suggested by the temperature trend series in the study area) [100,101,102,103,104]. Nevertheless, we cannot properly ascertain whether the observed declines are driven by any of the mentioned stressors with the present sampling design. Further research to assess the long-term temporal dynamics of saproxylic beetle assemblages is needed to more robustly relate changes in diversity and network patterns to global stressors and their additive effects.

### 4.2. Temporal Beta Diversity of the Tree Hollow–Saproxylic Beetle Interaction

This is the first attempt to disentangle the components of beta diversity of interactions for saproxylic beetle communities in Mediterranean forests. In spite of significant interannual changes in species composition, the beta diversity of interactions was affected most by interaction turnover. Ramos-Robles et al. [89] showed marked total interaction beta diversity (around 0.71) and a similar influence of species composition and interaction turnover on host tree–saproxylic Cerambycidae interaction networks in tropical dry forests. In our typically Mediterranean forest system, however, much lower total interaction beta diversity values were obtained (0.2 on the park scale and from 0.3 to 0.45 considering woodland types). Instead, interaction turnover was the main component explaining the interannual reorganization of the interactions in tree hollow–saproxylic beetle networks. This supports the notion that the beta diversity of interactions in saproxylic communities could be modeled by the constant reconnection of interactions across the pool of prevailing microhabitats (tree hollows) over time [105]. Thus, the interaction turnover would generate variation in the way that saproxylic species interact with woody resources (strict saproxylics) or with their prey (saproxylic predators). Furthermore, the reconnection of interactions was stronger in the sclerophyllous and riparian woodlands, which suggests that the tree hollows in those habitats are dissimilar in terms of interactions [79]. All these results indicate that temporal reconfigurations in the composition of suitable tree hollows promote contrasting responses in the interaction turnover across woodland types.

### 4.3. Saproxylic Communities’ Vulnerability in a Changing World

The general increase in saproxylic communities’ vulnerability strengthens the idea of alarming microhabitat suitability loss. Tree hollow–saproxylic beetle networks exhibited less vulnerability when random and directed extinctions of the least interconnected tree hollows were conducted, which means that most saproxylic species would survive even when a large proportion of tree hollows is removed. Nevertheless, the specialist beetles that depend on particular breeding microsites (differential interactions) would be more vulnerable [87,106]. In contrast, saproxylic communities crumbled quickly when the most interconnected tree hollows were first exterminated. All these results indicate that the bulk of beetle interactions occurred with the best-interconnected (densely and heterogeneously distributed interactions) suitable tree hollows across Mediterranean woodland types, which underlines their key role in modeling saproxylic communities’ complexity (diversity of species and interactions) and resilience [54]. Therefore, temporal changes in the composition of tree hollows may mediate complexity/stability dynamics in saproxylic communities.

In this regard, those insect communities that depend on specific microhabitats can substantially suffer from their alteration, reduction, or loss [20,42,104]. Temporal changes in structural trophic resources may unleash waves of secondary extinctions at the other trophic level [11,13,57], namely bottom-up trophic cascades. Thus, a continuous microhabitat modification, as is the case of suitable tree hollows, may extend from species loss to a wider simplification of natural communities [90,104,107]. For example, severe loss of insect diversity and abundance is expected to provoke cascading effects on food webs through closely interacting species or intermediate species [11,12,14,57] and could reduce prey availability for saproxylic predators. To summarize, both microhabitat loss and insect decline must be seen as triggering factors of secondary extinctions in saproxylic communities.

The recreation of potential threat scenarios based on the recorded interannual loss in microhabitat suitability across Mediterranean woodlands provided several pieces of evidence. First, core tree hollows consistently obtained some of the highest values for species degree and species strength, which reinforces the idea of the central role of suitable tree hollows in the assembly of saproxylic networks over time. Second, more complex distributions of core tree hollows were found when lower species strength values were considered, which highlights that relatively less connected tree hollows can also hold differential interaction arrangements. Third, no woodland type would present nestedness in the most critical microhabitat alteration scenarios, which would further endanger saproxylic communities’ stability. In such a hypothetic context, interaction complexity (connectance or linkage density) and, hence, stability would plummet, namely less diverse and less specialized (loss of nestedness) saproxylic communities leading to enhanced vulnerability to cascading extinctions [13,36,57,108,109,110]. This scenario is especially worrying in the sclerophyllous woodland and riparian woodlands. The network analysis procedures revealed that the saproxylic insect communities which inhabit tree hollow microhabitats in Mediterranean forests are facing an unprecedented temporal interaction decline that could even worsen if tree hollows become less suitable (i.e., because of dryness) in the current global change context.

## 5. Conclusions

We conclude that interaction decline was extensive across woodland types, which suggests that interannual variation is unlikely to have been caused by the typical fluctuations in diversity patterns of saproxylic communities (see [48]). Hence, temporal changes may be due to large-scale stress factors, such as climate change [2,15,48]. If current trends continue, even the pivotal role of saproxylic communities in ecosystems’ service delivery would be seriously compromised. To anticipate uncertain global change impacts, more efforts should be made to not only protect effective habitats and microhabitats for the conservation of saproxylic communities and their ecosystem services [18,19] but to also detect habitat vulnerability based on diversity [111] and interaction data. Therefore, network methods can provide enlightening information about ecosystem functioning and should be seen as valuable tools for identifying priority conservation areas and to further develop knowledge-based conservation measures and plans.

## Figures and Tables

**Table 1:** Network patterns and interaction attributes on the park scale and per woodland site for 2009–2010 and 2021–2022 and for the two potential threat scenarios based on (A) the transformation of qualitative tree hollow cores; (B) the transformation of quantitative tree hollow cores.

	Park Scale				Deciduous Woodland				Riparian Woodland				Sclerophyllous Woodland			
	2009	2021	A	B	2009	2021	A	B	2009	2021	A	B	2009	2021	A	B
**No. of** **species**	111	116	95	87	76	86	67	41	72	46	34	27	54	48	38	30
**No. of** **individuals**	2348	1680	1259	977	1222	790	444	376	618	569	142	133	508	321	290	277
***NODF***	15.75 *	10.63 *	9.29 *	9.40 *	26.70 *	19.62 *	17.52	20.64	23.69 *	18.18	13.89	15.12	19.70	14.02	14.23	17.49
***C***	0.11	0.07	0.07	0.07	0.20	0.14	0.14	0.17	0.18	0.14	0.11	0.12	0.15	0.11	0.11	0.14
***L/S***	3.66	2.30	2.05	2.00	2.66	1.87	1.77	1.93	2.32	1.59	1.16	1.17	1.90	1.40	1.28	1.44
***LD***	9.58	5.77	4.43	4.65	7.21	7.02	5.26	4.38	8.22	2.78	2.98	2.84	4.00	2.74	2.34	2.38
***IE***	0.63	0.53	0.50	0.54	0.64	0.60	0.63	0.63	0.68	0.42	0.55	0.54	0.54	0.43	0.40	0.40
***H2’***	0.36	0.54	0.57	0.56	0.38	0.53	0.51	0.47	0.36	0.65	0.66	0.66	0.51	0.68	0.69	0.62
***V-ratio***	8.85	4.62	4.79	5.03	3.67	1.77	1.87	2.00	3.24	0.97	0.54	0.60	2.60	1.73	2.18	2.65
**RR**	0.90	0.84	0.81	0.80	0.93	0.91	0.89	0.87	0.92	0.80	0.70	0.70	0.87	0.84	0.80	0.79
**RM**	0.61	0.54	0.54	0.52	0.69	0.73	0.73	0.66	0.67	0.65	0.49	0.48	0.70	0.70	0.57	0.52
**RL**	0.99	0.97	0.96	0.95	0.99	0.97	0.98	0.95	0.98	0.89	0.85	0.84	0.97	0.97	0.94	0.91

*NODF*: nestedness using the *NODF* estimator (*: significant values, *p* < 0.05); *C*: connectance; *L/S*: links per species; *LD*: linkage density; *IE*: interaction evenness; *H2′*: specialization index; V-ratio: variance ratio; RR: robustness for random tree hollow extinction; RM: robustness for directed extinction from the most to the least connected tree hollows; RM: robustness for directed extinction from the least to the most connected tree hollows.

**Table 2:** Beta diversity of the hollow–saproxylic interactions on the studied park and woodland scales.

	Park Scale	Deciduous	Riparian	Sclerophyllous
*β_S_*	0.758	0.691	0.667	0.681
*β_WN_*	0.163	0.225	0.237	0.338
*β_ST_*	−0.038	−0.065	−0.116	−0.104
*β_OS_*	0.201	0.290	0.352	0.442

## Data Availability

The data presented in this study are available upon request from the corresponding author or E.M.

## Supplementary Materials

The following is available online at https://www.mdpi.com/article/10.3390/insects14050446/s1, Table S1: Species list and abundance of saproxylic beetles in each woodland type in 2009–2010 and 2021–2022.
