# Supplementary material for: Interaction Networks Help to Infer the Vulnerability of the Saproxylic Beetle Communities That Inhabit Tree Hollows in Mediterranean Forests
Source: Insects. 2023 May 9;14(5):446. doi: 10.3390/insects14050446 (PMC10231080; doi:10.3390/insects14050446)
Supplement: Supplementary file 1 [file insects-14-00446-s001.zip › insects-2342845-supplementary.pdf]

Table S1 Species list and abundance of saproxylic beetles in each woodland type in 2009-2010 and 2021-2022.

Qp: *Quercus pyrenaica*, Fr: *Faxinus angustifolia*, Qi: *Quercus ilex*

		**2009/2010**			**2021/2022**			
**Family**	**Species**	**Qp**	**Fr**	**Qi**	**Qp**	**Fr**	**Qi**	**TOTAL**
Aderidae	*Aderus populneus*	14	7	3	6	0	0	30
	Cnopus minor	0	0	1	1	0	0	2
	Otolelus neglectus	0	0	1	0	0	0	1
Anthicidae	*Microhoria* cf. *fasciata*	0	0	0	1	0	0	1
	Microhoria sp.	0	0	1	0	0	0	1
Biphyllidae	*Diplocoelus fagi*	1	14	3	0	0	0	18
Brentidae	*Amorphocephala coronata*	0	0	0	2	0	0	2
Buprestidae	*Acmaeodera degener*	0	0	2	0	0	4	6
	Acmaeoderella cyanipennis	0	0	0	0	0	1	1
	Eurythyrea quercus	1	0	0	3	0	0	4
Carabidae	*Acupalpus cantabricus*	1	0	0	0	0	0	1
	Asaphidion stierlini	1	0	0	0	0	0	1
	Bembidion obtusum	0	0	0	0	1	0	1
	Bembidion tethys	3	0	0	0	0	0	3
	Brachinus sclopeta	0	0	1	0	0	0	1
	Calathus cinctus	0	0	0	0	1	0	1
	Calathus granatensis	260	79	236	42	240	196	1053
	Calathus mollis	0	0	0	0	1	0	1
	Calodromius bifasciatus	0	0	0	1	0	0	1
	Carabus lusitanicus	1	0	0	0	0	7	8
	Cryptophonus tenebrosus	0	0	1	0	0	0	1
	Leistus expansus	2	0	0	0	0	0	2
	Masoreus wetterhallii	0	0	1	0	0	0	1
	Notiophilus marginatus	0	0	0	1	0	0	1
	Porotachys bisulcatus	0	1	0	0	0	0	1
	Steropus globosus	1	0	4	0	0	0	5
	Syntomus foveatus	1	0	1	0	0	1	3
	Trechus obtusus	3	0	1	0	6	0	10
Cerambycidae	*Alocerus moesiacus*	2	0	7	1	0	4	14
	Cerambyx welensii	0	0	0	2	0	0	2
	Prinobius myardi	0	1	1	0	0	0	2
	Stictoleptura trisignata	5	1	6	2	2	3	19
Cetoniidae	*Cetonia aurataeformis*	96	65	21	7	9	2	200
	Protaetia cuprea	4	10	12	1	4	2	33
	Protaetia opaca	1	4	2	1	4	4	16
Ciidae	*Cis striatulus*	0	0	0	0	0	1	1
Clambidae	*Calyptomerus dubius*	0	1	0	1	2	0	4
Cleridae	*Opilo domesticus*	1	1	3	0	0	2	7
Corylophidae	*Arthrolips* sp.	0	0	0	1	0	0	1
Curculionidae	*Camptorhinus statua*	38	2	8	3	0	0	51
	Dryocoetes villosus	0	0	0	2	0	0	2
	Platypus cylindrus	0	0	1	1	0	0	2
	Xyleborinus saxesenii	6	13	2	0	0	0	21

	Xyleborus dryographus	1	3	0	3	0	0	7
	Xyleborus monographus	61	62	45	93	0	1	262
Dermestidae	*Anthrenus angustefasciatus*	0	0	0	0	0	1	1
	Anthrenus festivus	1	4	0	12	4	1	22
	Anthrenus minutus	0	21	1	0	0	1	23
	Anthrenus munroi	0	0	0	1	0	0	1
	Anthrenus verbasci	0	3	2	0	0	0	5
	Attagenus trifasciatus	0	7	2	0	1	2	12
	Dermestes bicolor	1	0	0	0	0	0	1
	Dermestes hispanicus	0	0	4	0	0	0	4
	Dermestes undulatus	0	0	0	0	0	1	1
	Orphilus niger	0	0	0	0	1	0	1
	Paranovelsis aequalis	0	0	2	7	0	0	9
	Thorictus cf grandicollis	0	0	0	2	0	0	2
	Trogoderma inclusum	0	0	0	0	1	0	1
Dynastidae	*Oryctes nasicornis*	0	0	0	0	0	1	1
Elateridae	*Ampedus aurilegulus*	20	18	0	6	2	0	46
	Athous obsoletus	0	0	0	2	0	0	2
	Cardiophorus signatus	0	0	0	5	2	1	8
	Cnemeplatia rufa	0	0	0	0	2	0	2
	Ectamenogonus montandoni	0	5	0	0	0	0	5
	Elater ferrugineus	11	7	0	4	1	0	23
	Elathous platiai	0	0	1	0	0	0	1
	Ischnodes sanguinicollis	19	13	0	0	2	0	34
	Lacon punctatus	2	4	0	2	4	0	12
	Limoniscus violaceus	0	1	0	0	1	0	2
	Megapenthes lugens	6	8	0	0	4	0	18
	Melanotus dichrous	0	0	0	0	0	2	2
	Procraerus tibialis	1	7	0	3	5	0	16
Endomychidae	*Cholovocera formicaria*	0	0	0	0	0	2	2
	Symbiotes gibberosus	1	2	0	1	2	0	6
Eucinetidae	*Nycteus meridionalis*	1	0	0	0	0	0	1
Histeridae	*Abraeus perpusillus*	9	7	0	7	0	0	23
	Aeletes atomarius	0	1	0	1	0	0	2
	Gnathoncus communis	22	12	0	9	0	3	46
	Gnathoncus nannetensis	8	4	0	47	5	1	65
	Hetaerius ferrugineus	1	0	0	5	0	1	7
	Kissister minimus	0	0	6	6	0	2	14
	Margarinotus merdarius	4	2	3	7	4	1	21
	Margarinotus uncostriatus	0	0	0	11	0	0	11
	Merohister ariasi	1	1	3	1	0	1	7
	Onthophilus sp.	0	0	0	0	0	7	7
	Paromalus flavicornis	22	3	0	4	2	0	31
	Platylomalus complanatus	0	1	0	0	0	0	1
	Platysoma filiforme	1	1	0	8	0	0	10
Laemophloeidae	*Cryptolestes ferrugineus*	1	0	1	1	0	0	3
	Laemophloeus nigricollis	0	1	0	0	0	0	1
	Placonotus testaceus	1	1	0	0	0	0	2
Lucanidae	*Dorcus parallelipipedus*	7	9	0	3	5	0	24

	Pseudolucanus barbarossa	0	0	0	1	0	0	1
Melandryidae	*Orchesia micans*	1	2	0	19	5	0	27
Melyridae	*Anthocomus fenestratus*	10	1	4	5	0	0	20
	Aplocnemus limbipennis	0	1	0	2	1	0	4
	Axinotarsus marginalis	0	0	4	0	0	2	6
	Falsomelyris andalusiaca	0	2	0	0	0	0	2
	Hypebaeus albifrons	2	0	0	1	0	0	3
	Mauroania bourgeoisi	1	0	1	3	0	1	6
	Troglops furcatus	18	5	9	5	7	1	45
Mycetophagidae	*Litargus connexus*	7	2	0	0	0	0	9
	Mycetophagus quadriguttatus	44	4	2	14	0	0	64
Nitidulidae	*Amphotis marginata*	1	0	0	0	0	0	1
	Carpophilus cf. *dimidiatus*	0	0	0	1	0	0	1
	Cryptarcha strigata	0	0	0	1	0	0	1
	Epuraea fuscicollis	95	0	5	76	2	3	181
	Soronia oblonga	56	3	2	46	6	4	117
Oedemeridae	*Ischnomera xanthoderes*	24	14	24	23	80	7	172
Ptinidae	*Dignomus irroratus*	0	1	0	2	0	0	3
	Dorcatoma agenjoi	7	0	0	2	0	1	10
	Mizodorcatoma dommeri	0	0	0	0	1	0	1
	Niptodes ferrugulus	0	0	0	5	0	0	5
	Oligomerus brunneus	6	1	0	10	0	0	17
	Ptinus bidens	5	1	1	10	3	0	20
	Ptinus hirticornis	1	0	1	1	3	1	7
	Ptinus obesus	0	0	0	0	0	1	1
	Ptinus pyrenaeus	0	0	0	1	0	0	1
	Ptinus spitzyi	0	0	0	5	0	0	5
	Ptinus timidus	159	55	6	13	6	2	241
	Rhamna semen	6	3	6	93	2	2	112
	Stagetus elongatus	0	2	0	0	0	0	2
	Stagetus micoae	0	1	0	5	1	3	10
Salpingidae	*Salpingus tapirus*	0	0	0	1	0	0	1
	Sphaeriestes reyi	0	0	0	0	0	3	3
Scirtidae	*Prionocyphon serricornis*	13	18	11	18	118	26	204
Scraptiidae	*Anaspis regimbarti*	9	1	0	0	0	0	10
	Scraptia testacea	13	25	9	0	0	0	47
Silvanidae	*Ahasverus advena*	1	0	0	0	0	0	1
	Airaphilus peyerimhoffi	0	0	0	1	0	1	2
	Oryzaephilus surinamensis	0	0	1	0	0	1	2
	Silvanus bidentatus	0	2	0	0	0	0	2
	Uleiota planata	4	1	0	0	0	0	5
Tenebrionidae	*Cnemeplatia rufa*	0	0	0	2	0	0	2
	Corticeus fasciatus	1	0	0	3	0	0	4
	Eledonoprius armatus	7	0	0	2	0	0	9
	Euboeus anthracinus	14	4	2	12	0	0	32
	Euboeus granulatus	0	0	1	1	0	2	4
	Isomira hispanica	0	1	8	0	1	2	12
	Mycetochara linearis	1	15	0	2	1	0	19
	Mycetochara quadrimaculata	13	27	20	40	7	2	109

	Palorus subderpressus	0	0	0	5	0	0	5
	Prionychus ater	0	0	0	1	0	0	1
	Prionychus fairmairei	4	8	0	0	1	0	13
	Pseudocistela ceramboides	13	3	0	10	0	0	26
	Stenohelops sublinearis	3	1	0	0	0	0	4
	Tenebrio cf. *codinae*	0	0	0	3	6	0	9
	Tenebrio punctipennis	24	4	0	0	0	0	28
Trogositidae	*Temnochila caerulea*	0	1	0	0	0	0	1
	Tenebroides maroccanus	0	1	2	0	0	0	3
Zopheridae	*Colydium elongatum*	4	1	0	10	0	0	15
	Endophloeus marcovichianus	11	0	0	4	0	0	15
	Synchita fallax	0	0	0	1	0	0	1
